# Glucose Absorption by the Bacillary Band of *Trichuris muris*

**DOI:** 10.1371/journal.pntd.0004971

**Published:** 2016-09-02

**Authors:** Tina V. A. Hansen, Michael Hansen, Peter Nejsum, Helena Mejer, Matthew Denwood, Stig M. Thamsborg

**Affiliations:** 1 Department of Veterinary Disease Biology, Faculty of Health and Medical Sciences, University of Copenhagen, Frederiksberg, Denmark; 2 Department of Plant and Environmental Sciences, Faculty of Science, University of Copenhagen, Frederiksberg, Denmark; 3 Department of Large Animal Sciences, Faculty of Health and Medical Sciences, University of Copenhagen, Frederiksberg, Denmark; Uniformed Services University of the Health Sciences, UNITED STATES

## Abstract

**Background:**

A common characteristic of *Trichuris* spp. infections in humans and animals is the variable but low efficacy of single-dose benzimidazoles currently used in mass drug administration programmes against human trichuriasis. The bacillary band, a specialised morphological structure of *Trichuris* spp., as well as the unique partly intracellular habitat of adult *Trichuris* spp. may affect drug absorption and perhaps contribute to the low drug accumulation in the worm. However, the exact function of the bacillary band is still unknown.

**Methodology:**

We studied the dependency of adult *Trichuris muris* on glucose and/or amino acids for survival *in vitro* and the absorptive function of the bacillary band. The viability of the worms was evaluated using a motility scale from 0 to 3, and the colorimetric assay Alamar Blue was utilised to measure the metabolic activity. The absorptive function of the bacillary band in living worms was explored using a fluorescent glucose analogue (6-NBDG) and confocal microscopy.

To study the absorptive function of the bacillary band in relation to 6-NBDG, the oral uptake was minimised or excluded by sealing the oral cavity with glue and agarose.

**Principal Findings:**

Glucose had a positive effect on both the motility (*p* < 0.001) and metabolic activity (*p* < 0.001) of *T*. *muris in vitro*, whereas this was not the case for amino acids. The 6-NBDG was observed in the pores of the bacillary band and within the stichocytes of the living worms, independent of oral sealing.

**Conclusions/Significance:**

*Trichuris muris* is dependent on glucose for viability *in vitro*, and the bacillary band has an absorptive function in relation to 6-NBDG, which accumulates within the stichocytes. The absorptive function of the bacillary band calls for an exploration of its possible role in the uptake of anthelmintics, and as a potential anthelmintic target relevant for future drug development.

## Author Summary

The human whipworm, *Trichuris trichiura* is prevalent in many tropical and subtropical countries and is believed to infect more than 460 million people worldwide. Treatment with single-dose albendazole or mebendazole is the current control strategy for human trichuriasis. This strategy, however, has a poor-to-mediocre treatment effect. The reason for the low treatment efficacy has been assessed in various ways, including genetic analysis, and both *in vitro* and *in vivo* pharmacological studies. However, these studies have not been conclusive and did not evaluate whether the biology of *Trichuris* spp. may have an impact on the inadequate treatment efficacy. To assess a possible reason for this, we here explore the absorptive function of a specialised structure, named the bacillary band. We found that glucose was absorbed by the band and accumulated within the worm independent of oral ingestion, and we speculate that anthelmintics may enter the worm by the same route. If this is the case, this new insight may be used to optimise drug formulations of current and/or future anthelmintic drugs in the treatment of human trichuriasis.

## Introduction

Whipworms, *Trichuris* spp., infect humans and a wide range of mammals throughout the world. The human whipworm, *T*. *trichiura*, is one of the major Soil Transmitted Helminths (STHs), and is estimated to infect 465 million people globally [1], mainly in tropical and subtropical regions, with the highest prevalence in Central Africa, southern India and Southeast Asia [2,3]. The benzimidazoles (BZs, i.e. albendazole (ALB) 400 mg and mebendazole (MBD) 500 mg are the most widely used anthelmintic drugs in mass drug administration (MDA) programmes against STHs targeting school-aged children [4]. A common characteristic of *Trichuris* spp. infections in humans and animals is a low to varied efficacy of a single dose of BZs [5–14]. It should be noted that mediocre to low treatment efficacy against *Trichuris* spp. infections in not only restricted to anthelmintics within the BZ drug class. Levamisole (LEV) and ivermectin (IVM), belonging to the imidazothiazoles derivatives and the macrocyclic lactones (ML) respectively, have shown low cure rates (CRs) of *T*. *trichiura* infections in humans [10,15,16] and low worm count reduction (WCR) of *T*. *suis* in pigs [17]. Even the relatively new amino-acetonitrile derivative, monepantel (MOP) has resulted in a *Trichuris* spp. WCR of between 0 and 16.3% in sheep [18], and was reported to have a moderate effect on *T*. *muris in vitro* and *in vivo* [11]. However, in recent randomised clinical trials, single-dose oxantel pamoate (20 mg/kg), a meta-oxyphenol analogue of pyrantel, has shown higher efficacy (CR: 26.3; 50%; ERR: 93.2; 97.7%) against *T*. *trichiura* infections [19,20], compared to ALB (CR: 2.6%; ERR: 45%) and MBD (CR: 9; 11.8%; ERR: 58.5; 75%). However, when oxantel pamoate is administered in combination with ALB, the efficacy (CR: 31.2%; ERR: 96%) [19,21] is similar to the results reported for ALB in combination with either MBD (CR: 46.1%; ERR: 93.2%) [22] or IVM (200 μg/kg) (CR: 37.9%; ERR: 91.1%), or IVM in combination with MBD (CR: 55.1%; ERR: 96.7%) [9]. In order to control STHs, the goals put forth by WHO are to reach 75% MDA coverage of preschool and school-aged children in need of treatment or from endemic countries [23]. However, the predicted impact of this goal on *T*. *trichiura* infections is limited due to the low efficacy of the drugs currently used [24]. Therefore, it is clear that other strategies against trichuriasis are required.

It is generally accepted that the main route of anthelmintic drugs into parasitic nematodes is by passive diffusion across the cuticle [25–27]. However, drug entry may be restricted due to the specialised morphological structures of *Trichuris* spp. (i.e. the bacillary band and the stichocytes), as well as the unique partly intracellular habitat of adult *Trichuris* spp. and the physiochemical properties of anthelmintic drugs at the location of the worm. These factors may result in a lower accumulation of anthelmintics in the worms, as shown in previous *in vitro* and *in vivo* studies from our laboratory [28,29]. The bacillary band is found in parasitic nematodes belonging to the order Trichinellida, also including *Trichinella* spp. and *Capillaria* spp. [30]. The band is located ventrally on the anterior two thirds of the thin thread-like part of *Trichuris* worms, where it covers about one third of the circumference [31]. This thin anterior part is embedded in the mucosa, creating a tunnel of epithelial cells, whereas the thicker posterior part of the body is free in the lumen of the caecum and proximal colon of the host [32–35], thus the habitat of adult *Trichuris* spp. is partly intracellular. The bacillary band consists of an area of cuticular pores, which is estimated to contain more than 50,000 pores in adult *T*. *muris*, and approximately 5,000 pores in the larval stages (L1- L2) [31]. Each pore overlies a bacillary cell which has a highly folded apical membrane, thus enlarging the surface area similar to absorptive cells found in organs such as the intestines or kidneys of mammals [31,36,37].

The exact feeding mechanism and the type of nutrients on which *Trichuris* spp. is dependent for survival are still debated. However, based on the mucous-rich environment, Jenkins (1970) concluded that *Trichuris* spp. ingests mucopolysaccharides and/or mucoproteins [34]. Some authors suggest that oral ingestion is the main route of entry [38], but as *Trichuris* spp. lack a muscular pharynx [39], it is doubtful whether this parasitic nematode is able to pump nutrients through the gut. A recent transcriptome analysis of the anterior part of *T*. *muris* revealed a relatively high expression level of chymotrypsin A-like serine proteases [40]. The authors proposed that these digestive enzymes were excreted to break down nutrients, which could subsequently be absorbed through the pores of the bacillary band. Other authors have proposed similar suggestions [31,34], but the function of the bacillary band remains unknown.

We believe that the morphology of the bacillary cells, the lack of a muscular pharynx of *T*. *muris* and the close proximity of the bacillary band to the cytosol of the intestinal epithelial cells of the host suggest that this organ is involved in nutrient absorption. We therefore hypothesised that adult *T*. *muris* absorb nutrients (glucose and/or amino acids) found in the surrounding micro-environment through the bacillary band. The aim of this study was therefore to assess whether *T*. *muris* is dependent on glucose and/or amino acids for survival *in vitro*, and whether glucose enters through the oral cavity and/or the bacillary band.

## Methods

### Ethics statement

The study was approved by the Experimental Animal Unit, University of Copenhagen (Denmark) based on national regulations from the Danish Animal Experiments Inspectorate (permission no. 2010/561-1914) and carried out in accordance with their guidelines.

### Chemicals, media and fluorophores

For the Alamar Blue assay, resazurin sodium salt (Sigma-Aldrich, DK) was dissolved in dH_2_O (125 mg/L), aliquoted into 10 mL tubes and kept at -20°C until use. Modified RPMI 1640 medium (without amino acids and glucose) from US Biological Life Sciences (US) was prepared according to the manufacturer’s recommendations with only minor modifications. In brief, 7.4 g modified RPMI 1640 powder was dissolved in 900 mL MilliQ water, followed by the addition of 2.0 g sodium bicarbonate, and the pH was adjusted to 7.1 at 21°C using HCl (1M). MilliQ water was added to make a final volume of 1 L. The RPMI media were filter-sterilised using a 0.2 μm membrane (Advantec MFS, Inc, US), stored at 4°C and used within 4 weeks. The RPMI medium without amino acids but with glucose was prepared from the medium described above by adding 1 g D-(+)-glucose (Sigma-Aldrich, DK) to a final concentration of 1 g/L (0.1%). RPMI 1640 media with amino acids and with or without glucose were purchased from Life Technologies (DK), and Hanks Balanced Salt Solution (HBSS) was purchased from Invitrogen (DK). All media used for the isolation and incubation of worms were supplemented with 1% (v/v) amphotericin B-penicillin-streptomycin solution (10,000 U/mL penicillin, 10,000 μg/mL streptomycin, 25 μg/mL amphotericin B; Life Technologies, DK). The concavalin A (Con-A) conjugate Alexa Fluor was dissolved in 0.1M sodium bicarbonate (5 mg/mL) pH 8.3, aliquoted into Eppendorf tubes and stored at -20°C until use. Stock solution of the glucose analogue 6-(*N*-(7-Nitrobenz-2-oxa-1,3-diazol-4yl)amino)-6-Deoxyglucose (6NBDG) was prepared by dissolving 6-NBDG in 100% dimethyl sulfoxide (DMSO) to a final concentration of 100 mM. The highest concentration of DMSO used in the experiments was 0.2% (v/v). Both fluorophores were purchased from Life Technologies, DK.

### Experimental animals and infection

Five 4-week-old female mice (C57BL/10) were purchased from Harlan Laboratories (Blackthorn, UK) once per week for 3 weeks (n = 15). Similarly, five 4-week-old female mice (C57BL/6) were purchased on two occasions from Taconic Biosciences (Lille Skensved, DK). All animals were acclimatised for 1 week prior to infection with 200 embryonated *T*. *muris* eggs given by oral gavage. The mice were kept in a 12-hour light/dark cycle in groups of five animals per cage (Scanbur 1291), where they had access to water and rodent pellets (Altromin 1324, Brogaarden, DK) *ad libitum*. To delay worm expulsion, the mice were given 1 mg/L dexamethasone (Dexsol, 2 mg/5 mL Oral solution, Rosemont Pharmaceuticals Ltd, UK) in the drinking water from 2 days before infection and onwards.

### Study design

This study includes three experiments: 1) an evaluation of the dependence of adult *T*. *muris* on glucose and amino acid *in vitro*; 2) a visualisation of the bacillary band, accumulation of 6-NBDG in the pores of the band, and absorption into the worm; 3) the absorption of 6-NBDG through the bacillary band and into the worms with sealed oral cavities. The experiment evaluating glucose and amino acid dependence was repeated three times with worms isolated from the C57BL/10 mice. Worms from the same mice were used for initial tests, visualisation of the bacillary band, accumulation of 6-NBDG in the pores of the band and absorption into the worms. In order to confirm accumulation and absorption of 6-NBDG into *T*. *muris* this part of the study was repeated three times. Worms isolated from C57BL/6 mice were used for the live uptake of 6-NBDG through the bacillary band and the evaluation of 6-NBDG absorption in worms with a sealed oral cavity; these experiments were repeated twice.

### Recovery of *Trichuris muris*

Infection was confirmed in all mice by a modified McMaster method from day 30 until day 35 when the mice were euthanised by cervical dislocation and eviscerated. The large intestine and caecum from each animal were dissected and placed in a petri dish containing saline at 37°C (0.9% NaCl). Using a stereo microscope, the intestine was opened longitudinally with a small scissor and the adult worms were gently lifted with curved forceps into 37°C saline (0.9%). Worms were washed using the following procedure: four times for 15 minutes in 37°C HBSS, followed by four times for 60 minutes in 37°C RPMI-1640 medium (with amino acids and 0.1% glucose). All isolated worms were mixed and randomly allocated to different types of media.

### *In vitro* evaluation of glucose and amino acid dependence

To test if glucose (glu) and amino acid (aa) are essential for *T*. *muris*, worm viability was evaluated after incubation in RPMI 1640 which differed only in the content of amino acids and glucose (0.1%). The composition of the four types of media were as follows: 1) RPMI +aa, +glu, 2) RPMI-aa, -glu, 3) RPMI +aa, -glu, 4) RPMI -aa, +glu. For each type of media, ten adult worms were incubated for 17 and 41 hours at 37°C (5% CO_2_, 21% O_2_, 90% relative humidity). Due to previous experience with *in vitro* incubation of *Trichuris* spp. in our laboratory, the incubation times were initially set to 24 and 48 hours. The experiment initially included worms with a glued oral cavity, but the glue process of 240 worms was less successful. Therefore, due to practical reasons, the incubation times were set to 17 and 41 hours. All incubations were made in triplicate, and therefore a total of thirty worms were included for each medium.

#### Assessment of *Trichuris muris* viability

The viability was evaluated by two methods: 1) visual motility evaluation by stereo microscopy, and 2) Alamar Blue, as previously described for viability evaluation of *T*. *muris* [41]. The motility evaluation was blinded and graded as follows: 3: normal motility (movement of the whole body), 2: low motility (slower movement of the whole body), 1: very low motility (movement of the thin anterior part only), 0: no movement for 10 seconds. For each media, ten random worms were scored using a stereomicroscope at 6.3x magnification. In the Alamar Blue assay, two random worms from each media were transferred into 1 mL of fresh 37°C RPMI media of the same type, supplemented with antibiotics and fungicide as described above. Following this, 50 μL of resazurin was added and the worms were incubated for another 6 hours. RPMI media (+glu, +aa) and resazurin with no worms added was included as a control. Following incubation, 200 μL of the media was transferred into black 96-well plates, and the fluorescent emission of the reduced resazurin was measured at 590 nm using a spectrofluormeter (SpectraMax Gemini XS, Molecular Devices, US) at 37°C. All resazurin measurements were made in duplicate.

### Labelling of bacillary pores and 6-NBDG absorption

To visualise the bacillary band, the Con-A conjugate Alexa Fluor 633 was used, as Con-A has previously been described to bind to glucose- or mannose-containing polysaccharides in the pores of the bacillary band of *T*. *muris* [31]. Final concentrations of 25, 50, 100 and 200 μg/mL were initially tested after 15, 30 and 45 minutes of incubation. Incubating the worms at 37°C RPMI media (without glucose) for 30 minutes at a final concentration of 100 μg/mL was found to yield optimal labelling of the bacillary pores with Alexa Fluor 633. Subsequent experiments were therefore performed using this concentration and incubation time. After incubation, excess Alexa Fluor 633 was removed by gently washing the worms in 10 mL RPMI media (without glucose).

D-glucose inhibits the uptake of NBDGs (i.e. 6-NBDG and 2-NBDG) in bacteria and mammalian cells [42,43], so RPMI medium devoid of glucose was used during the labelling and visualisation of 6-NBDG absorption. The glucose analogue 6-NBDG was chosen because this analogue is not phosphorylated by hexokinase and subsequently degraded to a non-fluorescent compound as 2-NBDG [44]. To optimise 6-NBDG exposure time, the worms were initially incubated for 5, 10 and 15 minutes in RPMI (without glucose) containing a final concentration of 200 μM 6-NBDG as previously described for the free-living nematode *Caenohabditis elegans* [45]. However, it became clear that the *T*. *muris* glucose absorption was rapid (under 10 minutes). To slow down the absorption rate of 6-NBDG, the glucose analogue was mixed with agarose (1%) to a final concentration of 200 μM, in which the thin anterior part (containing the bacillary band) was embedded. The living worms were embedded in the mixture using curved forceps to gently lift the worm at the thick posterior part, and the thin part of the worm was subsequently stretched by pulling it through a drop of agarose (40°C, 20 μL) placed on a preheated (40°C) glass slide. Another preheated glass slide was gently placed on top of the worm, so that the whole anterior thin part became embedded in the agarose-6-NBDG mixture. RPMI (without glucose) was added between the slides to keep the worm moist. The integrity of the cuticle was microscopically confirmed before the experiments, and movement of the posterior part of the worm (which was not fixed in agarose) was used as an indicator of viability. This method was used to visualise the accumulation of 6-NBDG in the pores of the bacillary band and to further investigate whether 6-NBDG enters the worms.

In order to follow the live uptake of 6-NBDG through the bacillary band, a continuous time series of the 6-NBDG absorption in living *T*. *muris* was recorded over a period of 20 minutes. The anterior part of the worm was fixed in agarose devoid of 6-NBDG. A liquid column of RPMI (without 6-NBDG) was created between water dipping objectives and the worm, and was subsequently exchanged with 6-NBDG containing RPMI using a suction device. The live-uptake part of the study included a total of two test worms and two control worms.

Confocal images were taken with a Leica SPX5 Confocal Laser Scanning Microscope using 40x (Leica, HCX APO L U-V-I 40x, 0.80W) and 63x objectives (Leica, HCX PL APO 63x, 1.20W) for videos and images, respectively. All images were taken with identical settings and subsequently optimised for brightness and contrast. For comparison, each image included in a time series was optimised equally. Prior to the study, only weak autofluorescence was detected in three *T*. *muris* (in particular in the oral cavity and the bacillary band) at excitation and emission wavelengths used for Alexa Fluor 633 (Ex: 632 nm, Em: 642–700 nm) and 6-NBDG (Ex: 471 nm, Em: 500–600 nm) using 63x objectives.

### Sealing of the oral cavity

To exclude oral uptake of 6-NBDG, the anterior tip of five *T*. *muris* were dipped two times in surgical glue (Histoacryl, B. Braun Surgical, S.A., Spain) with alternating exposure to RPMI media. This was done because the cyanoacrylate-based glue polymerises within seconds when exposed to water-containing substances. Between each Histoacryl-RPMI exposure, the glue was allowed to dry for 30 seconds, as recommended by the manufacturer. To further inhibit the exposure of the oral cavity to 6-NBDG, the outermost anterior part of the *T*. *muris* was embedded in agarose (1%) without 6-NBDG, which was allowed to solidify before the rest of the worm was embedded in agarose (1%) with 6-NBDG as described below. The glue has previously been used to seal the oral cavity of earthworms (*Lumbricus rubellus*) [46]. However, the potentially toxic effect of the glue on the cuticle and viability of parasitic nematodes, and the effect of the RPMI media on the glue, was initially tested on five adult *Ascaris suum* worms, chosen for practical reasons due to their large size. These worms were kept for 7 days following oral sealing under *in vitro* conditions equivalent to those described for *T*. *muris*, i.e., 37°C (5% CO_2_, 21% O_2_, 90% relative humidity). Five *A*. *suum* without oral sealing were included as controls. The sealed worms were evaluated daily for motility (compared to control worms, glue-cap attachment, and damage of the cuticle near the glue using a stereo microscope (Leica Wild M3Z). At day 7, the worms were sacrificed in ethanol (70%), the glue-caps separated from the worms with forceps and examined for visible holes using a stereo microscope.

### Statistical analysis

The motility and fluorescence data were analysed separately, using similar multivariate (generalised) linear mixed modelling approaches as follows.

Arithmetic means (± SD) of motility and the fluorescent emission from the Alamar Blue assay were calculated from three repeated experiments. For the motility data, three different cut-off points were used to classify the data into a binary response for logistic regression. Three separate analyses were necessary because the motility data showed a pronounced difference between worms incubated in RPMI with and without glucose (with glucose the motility score was nearly always 3, without glucose the motility score was nearly always 0), masking the relatively subtle effect of the amino acids. The first analysis compared all observations of between 0–1 to those between 2–3, with fixed effects describing the presence of glucose and presence of amino acids (an interaction with time could not be fitted because the data were almost totally separated by the presence or absence of glucose). The second analysis compared observations of 0-2 to observations of 3 for the RPMI with glucose present data only, with fixed effects describing the presence of amino acids and the time point (17 or 41 hours incubation). The third analysis compared observations of 0 to those of 1–3 for the RPMI (without glucose) data only, with the same fixed effects describing the presence of amino acids and the time point.

For the fluorescence data (Alamar blue), a single linear mixed model was used with fixed effects describing the presence of glucose, presence of amino acids, time point, and the interaction between presence of glucose and amino acids. A Box-Cox transformation was used for the response variable in order to improve the normality of the model residuals.

A random effect of the replicate number (between 1 and 12) was used in all models to control for repeated observations within the same petri dish. All analyses were performed in R [47] using the lme4 [48] and lmerTest [49] packages, and using the Box-Cox transformations implemented in the car [50] package.

## Results

### Viability of *T*. *muris*

**Fig 1:**
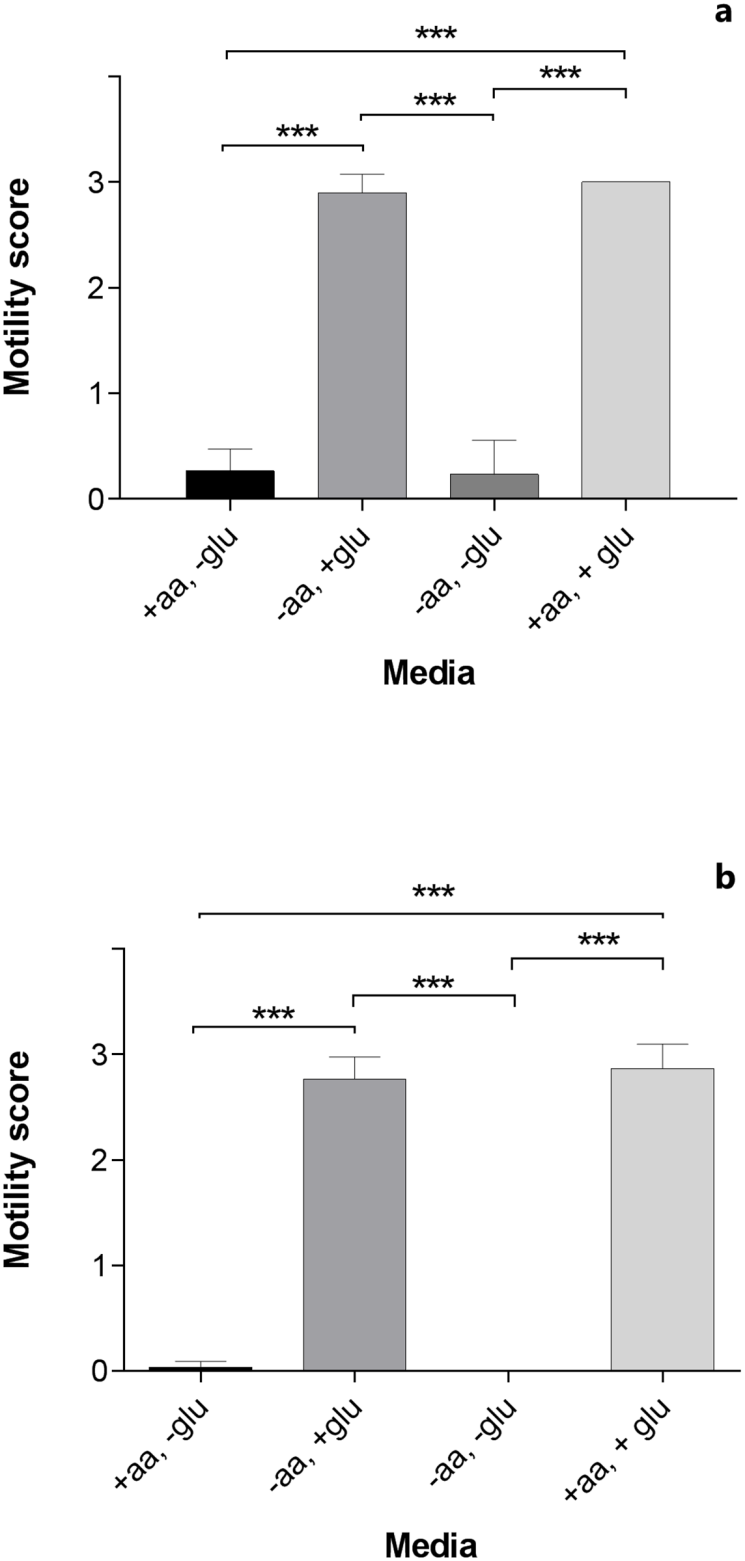
*In vitro* motility assay with adult *Trichuris muris*. The motility score is given as arithmetic means (± SD) of three individual studies where worms (n = 30) were incubated in RPMI medium with (+) or without (-) glucose (glu) and/or amino acids (aa) for 17 (Fig 1a) or 41 (Fig 1b) hours. ***: *p* < 0.001. No variation in motility was observed for worms incubated in +aa, +glu.

The motility score of *T*. *muris* incubated for 17 and 41 hours in RPMI media with or without glucose and/or amino acids is presented as the arithmetic means (± SD) of three experiments in Fig 1. Worms incubated in RPMI with glucose were significantly more motile (*p* < 0.001) than worms exposed to media devoid of glucose. Looking at dichotomized glucose data only (scores 0–2 vs. 3), time (17 vs. 41 hours) had a significant negative effect on worm motility (*p* = 0.037). A significant negative effect of time on worm motility (score 0 vs. 1–3) was also found for data without glucose (*p* = 0.004), but the magnitude of the difference between time points was substantially smaller than that of the presence compared to absence of glucose. No significant difference was found in the motility of worms incubated in media with amino acids compared to media without amino acids.

**Fig 2:**
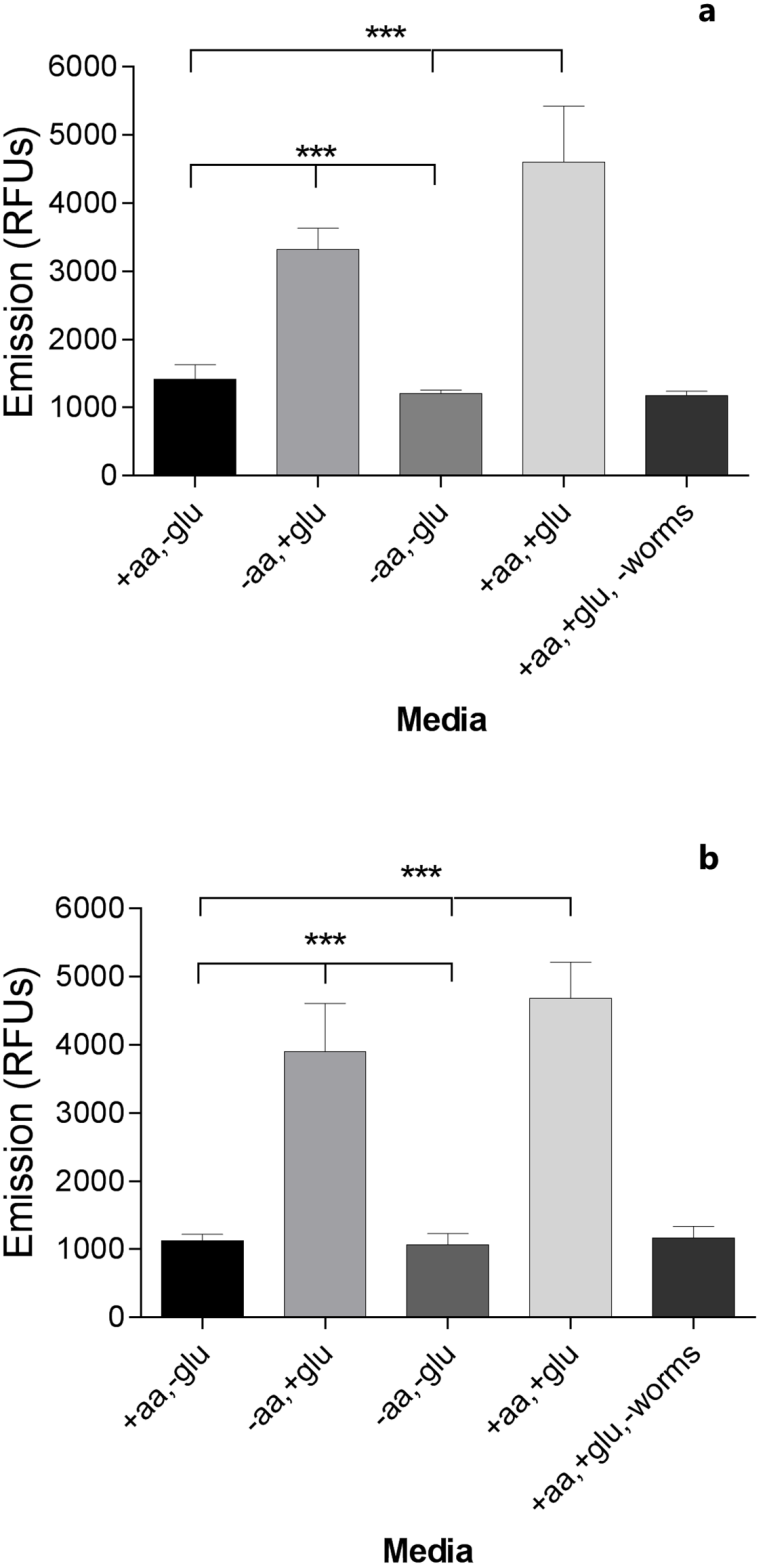
*In vitro* Alamar Blue assay with adult *Trichuris muris*. Emission of the reduced dye resorufin is given as arithmetic means (± SD) of three individual studies where worms (n = 4) were incubated in RPMI medium with (+) or without (-) glucose (glu) and/or amino acids (aa) for 17 (Fig 2a) or 41 (Fig 2b) hours.

The Alamar Blue assay (Fig 2 and Table 1) also showed that worm metabolism is glucose dependent, as demonstrated by the resorufin formed by worms incubated in RPMI with glucose being significantly higher than for worms incubated in media devoid of glucose (*p* < 0.001). In contrast, there was no significant effect of amino acids (*p* = 0.122), nor any significant effect of time on the metabolism of *T*. *muris* (*p* = 0.299). However, if the non-significant interaction and time terms are removed from the model, a significantly positive effect of amino acids is found (*p* = 0.005), with a corresponding magnitude of effect equal to 0.182 (approximately 15% of the magnitude of effect of glucose within the same model).

**Table 1:** *In vitro* Alamar Blue assay. Estimated effects of glucose, amino acids and time on the viability of *Trichuris muris* using a linear mixed model (with replicate as a random effect).

	Fixed Effect Estimate	Std. Error	*p*-value
**Glucose (presence vs. absence)**	1.149	0.064	< 0.001
**Amino acids (presence vs. absence)**	0.110	0.064	0.122
**Time Point (41 vs. 17 hours)**	-0.046	0.043	0.299
**Glucose: AA (interaction)**	0.144	0.090	0.150

### Absorption of 6-NBDG through the bacillary band

**Fig 3:**
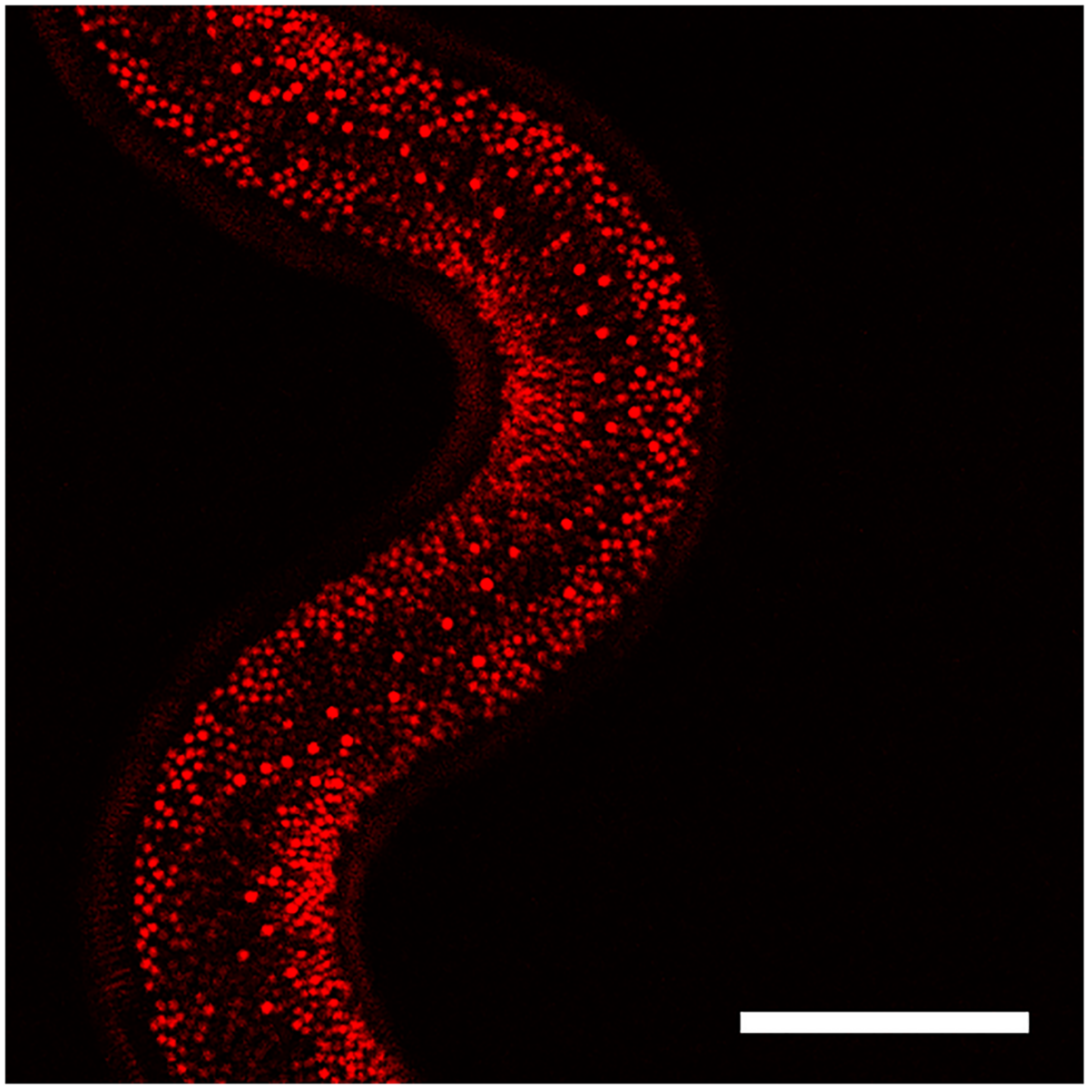
Confocal image showing the ventral side of the thin anterior part of an adult *Trichuris muris*. The bacillary band was labelled with Alexa Fluor 633 (100 μg/mL) for 30 minutes and can be seen as red circles or dots. Scale bar: 100 μm.

**Fig 4:**
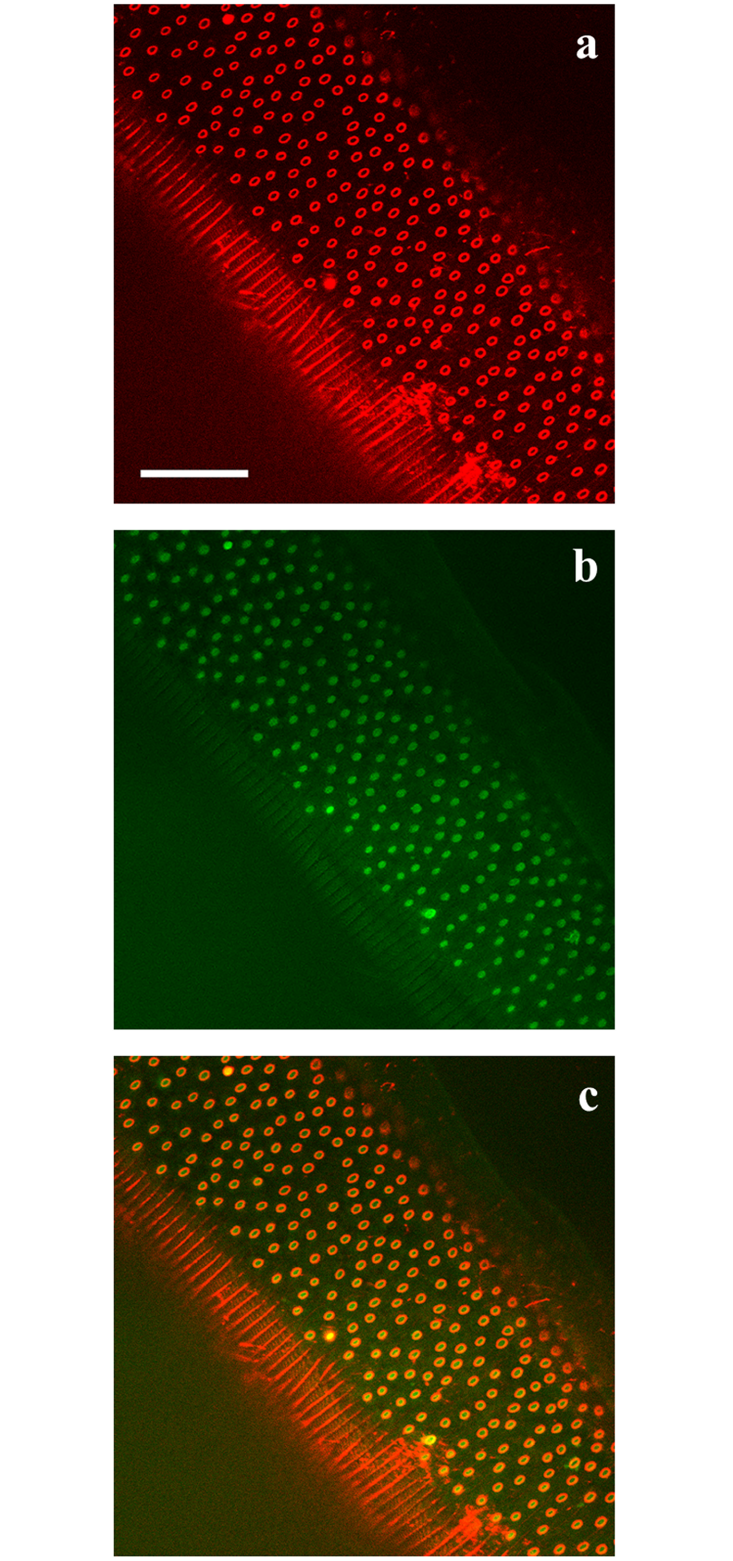
Confocal image of the ventral side of an adult *Trichuris muris* showing the pores of the bacillary band (Fig 4a) and accumulated 6-(*N*-(7-Nitrobenz-2-oxa-1,3-diazol-4-yl)amino)-6-Deoxyglucose (6-NBDG) (Fig 4b) in the pores of the bacillary band (Fig 4c) after incubation in agarose (1%) with 6-NBDG (200 μM) for approximately 5 minutes. Fluorescence from 6-NBDG and Alexa Fluor 633 is shown in green and red respectively. Scale bar: 30 μm.

**Fig 5:**
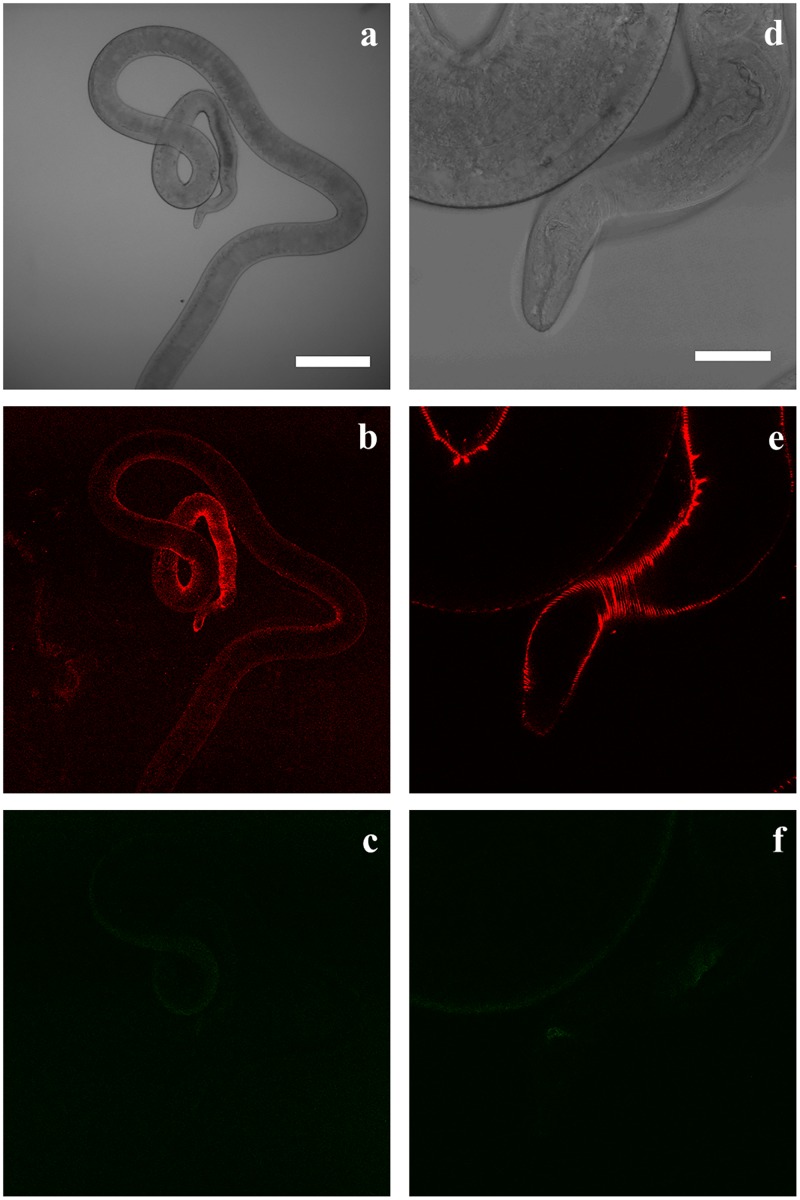
Transmission images (Fig 5a and d) and confocal section through the thin anterior part (Fig 5b and c) and oral cavity (Fig 5e and f) of an adult *Trichuris muris* not exposed to 6-(*N*-(7-Nitrobenz-2-oxa-1,3-diazol-4-yl)amino)-6-Deoxyglucose (6-NBDG) is shown for emission wavelengths between 500–600 (6-NBDG, Fig 5c and f) and 642–700 nm (Alexa Fluor 633, Fig 5b and e).

Fig 3 shows a ventral view of the bacillary band labelled with Alexa Fluor 633 for 30 minutes at a final concentration of 100 μg/mL. When worms were exposed to 6-NBDG, the glucose analogue accumulated in the pores of the band (Fig 4a–4c) and deeper into the worm tissue as shown in a confocal stack through the bacillary band (S1 Video). In worms not exposed to 6-NBDG, almost no autofluorescence was detected between 500–600 nm in (Fig 5a–5f). The dynamic process of 6-NBDG absorption was documented by making a time series of the bacillary band before, during and after the addition of 6-NBDG (200 μM) (S2 Video and S1 Fig). A lateral view of the bacillary band is shown in S2 Video, where it can be observed that 6-NBDG is absorbed, as the band becomes visually broader and the mean grey values of a region of the band increase from 1.7 to 5.8 during the 20-minute time period. However, autofluorescence of the bacillary band was observed before the addition of 6-NBDG, but disappeared abruptly when 6-NBDG containing RPMI media was added. Similarly, autofluorescence of the bacillary band was observed in the control worms, but did not disappear when RPMI media devoid of 6-NBDG was added (S3 Video and S2 Fig). In the test and control worms, the autofluorescence was weak and restricted to a narrow stripe in the bacillary band, which remained at a constant size in the control worms, and even had a small decrease in the mean grey value from 4.2 to 3.4 during the experiment.

**Fig 6:**
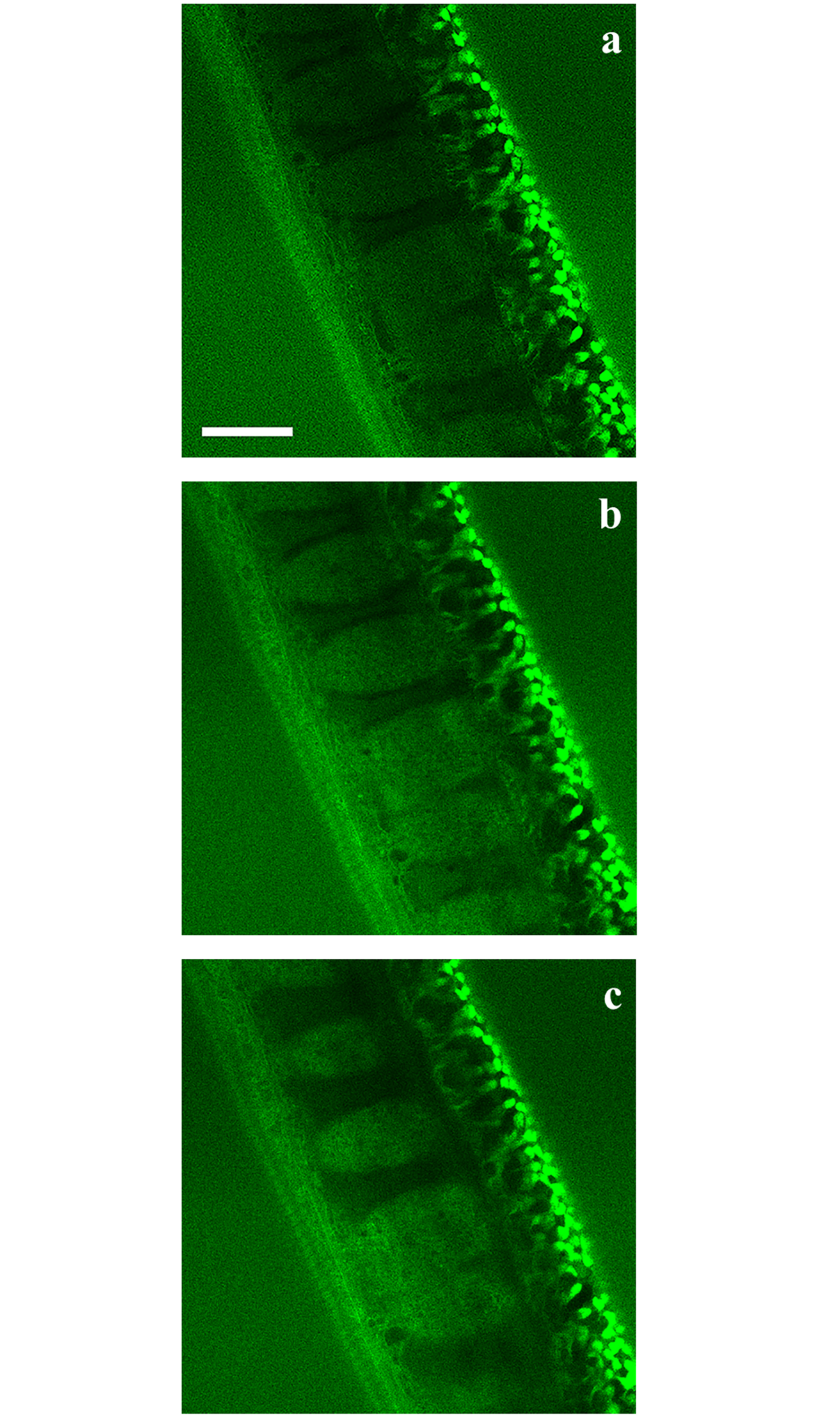
Confocal section through the thin anterior part of an adult *Trichuris muris* after incubation in agarose (1%) with 6-(*N*-(7-Nitrobenz-2-oxa-1,3-diazol-4-yl)amino)-6-Deoxyglucose (6-NBDG) (200 μM) for 6 (Fig 6a), 15 (Fig 6b) and 30 (Fig 6c) minutes. The glucose analogue 6-NBDG was absorbed by the bacillary band and accumulated within the stichocytes in which the mean grey value increased from 8.7 (Fig 6a) to 12.8 (Fig 6b) and 13.5 (Fig 6c). Scale bar: 30 μm.

**Fig 7:**
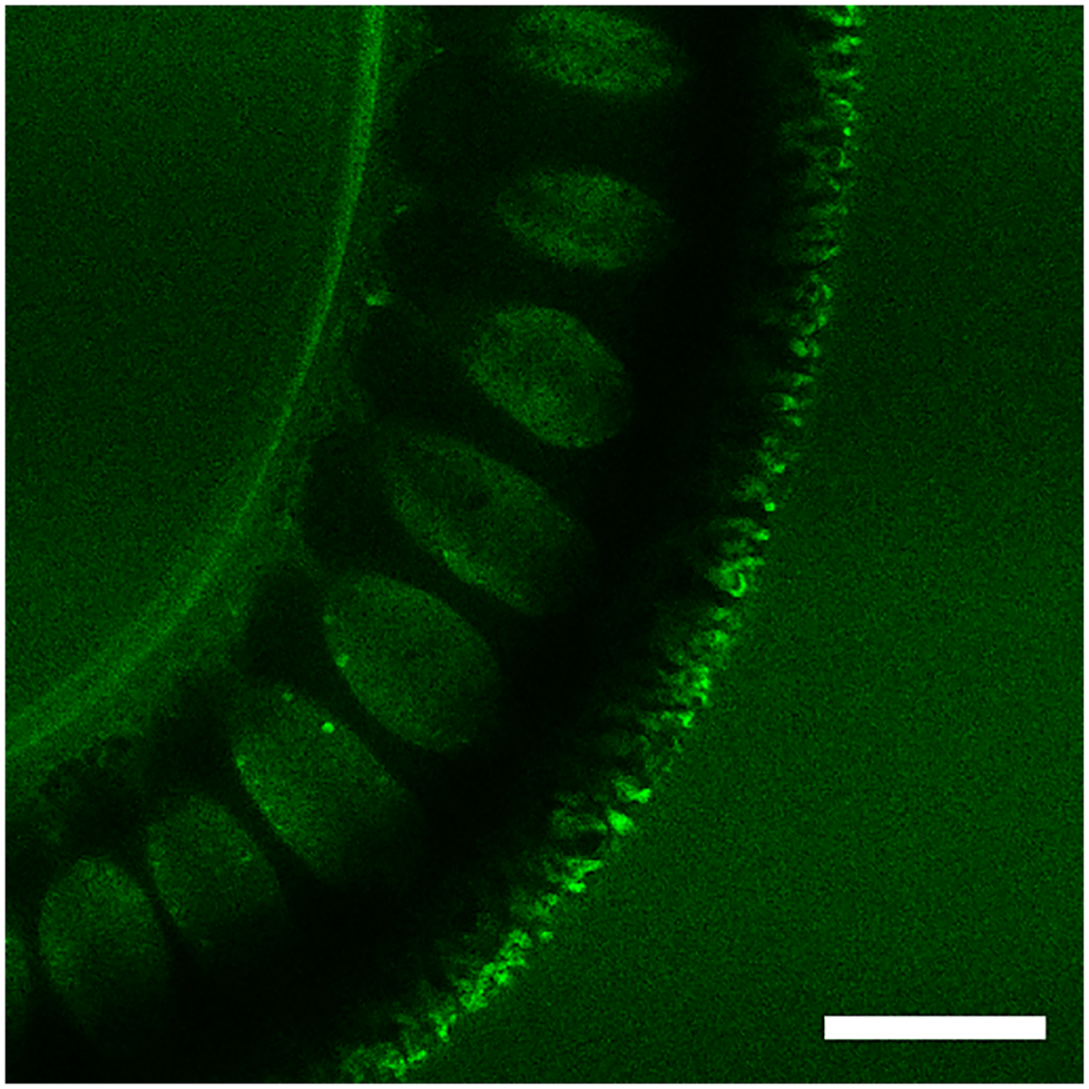
Confocal section through the thin anterior part of an adult *Trichuris muris* showing the accumulation of 6-(*N*-(7-Nitrobenz-2-oxa-1,3-diazol-4-yl)amino)-6-Deoxyglucose (6-NBDG) within the bacillary band, the stichocytes and the connecting membranes after incubation in agarose (1%) with 6-NBDG (200 μM) for approximately 30 minutes. Scale bar: 50 μm.

**Fig 8:**
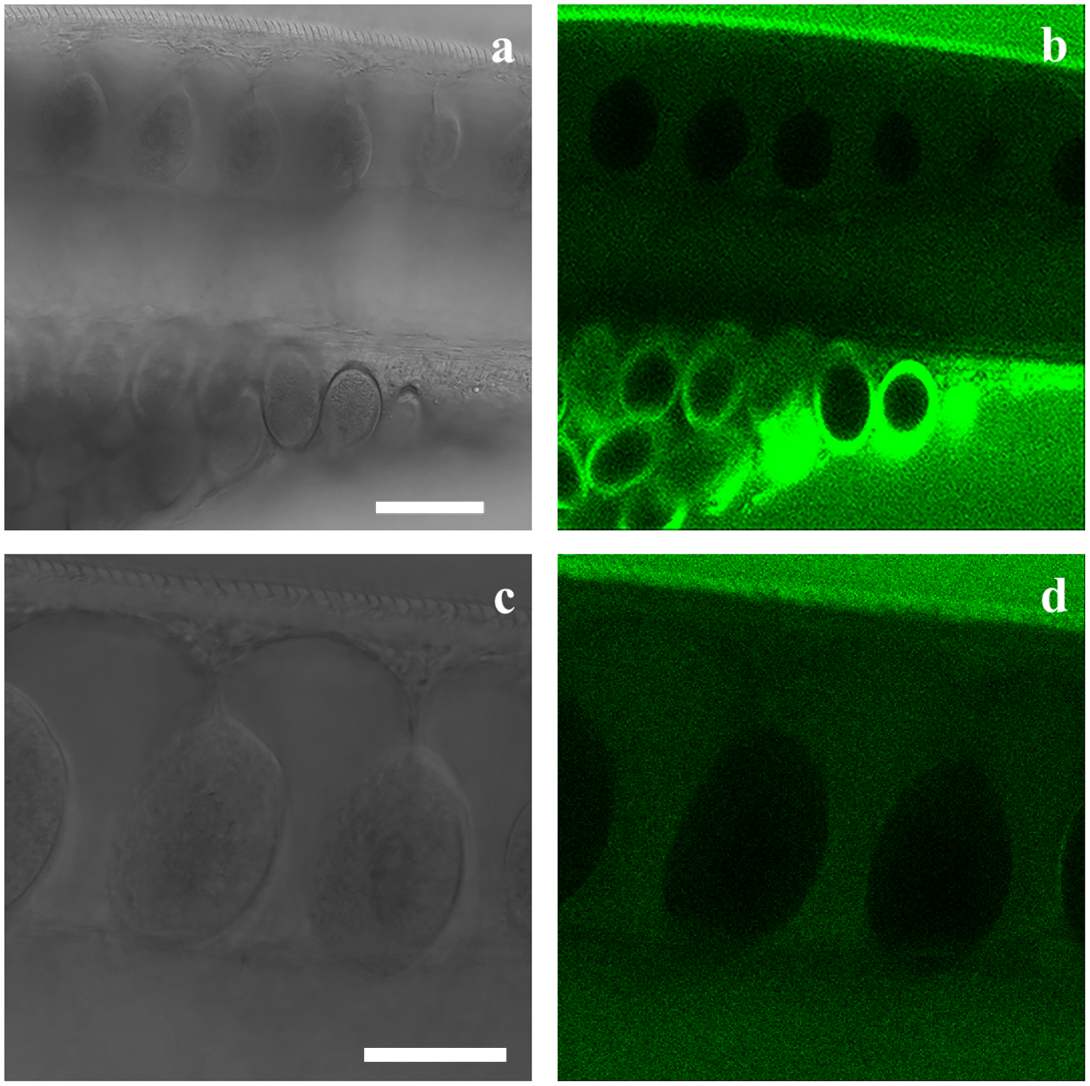
Transmission image (Fig 8a and 8c) and confocal section (Fig 8b and 8d) through an adult *Trichuris muris* at the junction between the thin anterior and the thick posterior part of the worm after incubation in agarose (1%) with 6-(*N*-(7-Nitrobenz-2-oxa-1,3-diazol-4-yl)amino)-6-Deoxyglucose (6-NBDG) (200 μM) for approximately 40 minutes. Note the eggs are located ventrally near the vulva, which is itself located at the border between the thin anterior and the thick posterior part of the worm (Fig 8a and 8b). The bacillary band was not visible in this area and the stichocytes and the connective membranes were devoid of 6-NBDG. Scale bar: 50 μm. High magnification of the stichocytes and the connective membranes devoid of 6-NBDG (Fig 8c and 8d). Scale bar: 30 μm.

To further investigate whether 6-NBDG enters the worms, consecutive pictures were taken of the bacillary band and the interior of the worms during a 30-minute incubation period with an interval of 1 minute and starting at 6 minutes post incubation. Fig 6 shows the accumulation of 6-NBDG in the bacillary band and the stichocytes at 6 minutes (Fig 6a), 15 min (Fig 6b) and 30 minutes (Fig 6c) post incubation. During this period, the intensity of 6-NBDG increased from 8.7 to 12.8 and 13.5 in mean grey values, showing the gradual accumulation of 6-NBDG within the stichocytes. The stichocytes appear to be connected to the bacillary band by membranes which also accumulate 6-NBDG (Fig 7), suggesting that 6-NBDG is transported from the bacillary band to the stichocytes via these membranous structures. Interestingly, at the junction between the oesophagus and the intestine, where the bacillary band could not be seen, the stichocytes were devoid of glucose (Fig 8a–8d). In Fig 8a and 8b, eggs are seen ventrally near the vulva, indicating the border between the thin anterior and the thicker posterior part of the worm. However, whether 6-NBDG passes from the bacillary band through the connecting membranes to the stichocytes, or is ingested through the oral cavity where it passes from the oesophagus to the stichocytes, could not be determined from this part of the study.

### Sealing of the oral cavity and glucose absorption by *T*. *muris*

**Fig 9:**
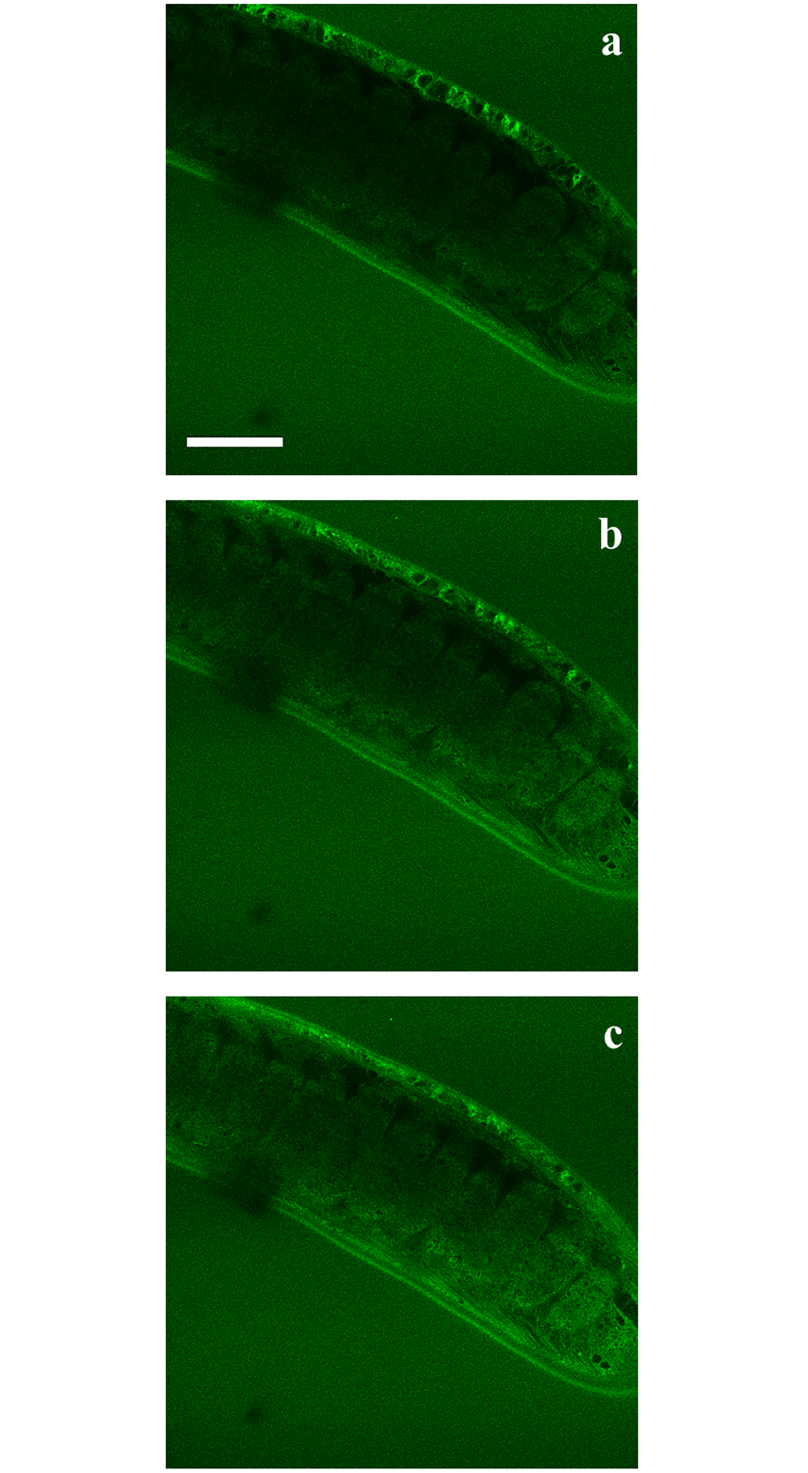
Confocal section through the thin anterior part of an adult *Trichuris muris* with sealed oral cavity after incubation in agarose (1%) with 6-(*N*-(7-Nitrobenz-2-oxa-1,3-diazol-4-yl)amino)-6-Deoxyglucose (6-NBDG) (200 μM) for 29 (Fig 9a), 44 (Fig 9b) and 56 (Fig 9c) minutes. Despite the sealed oral cavity, 6-NBDG accumulated within the stichocytes with mean grey values increasing from 5.8 to 8.1 and 9.4. Scale bar: 50 μm.

**Fig 10:**
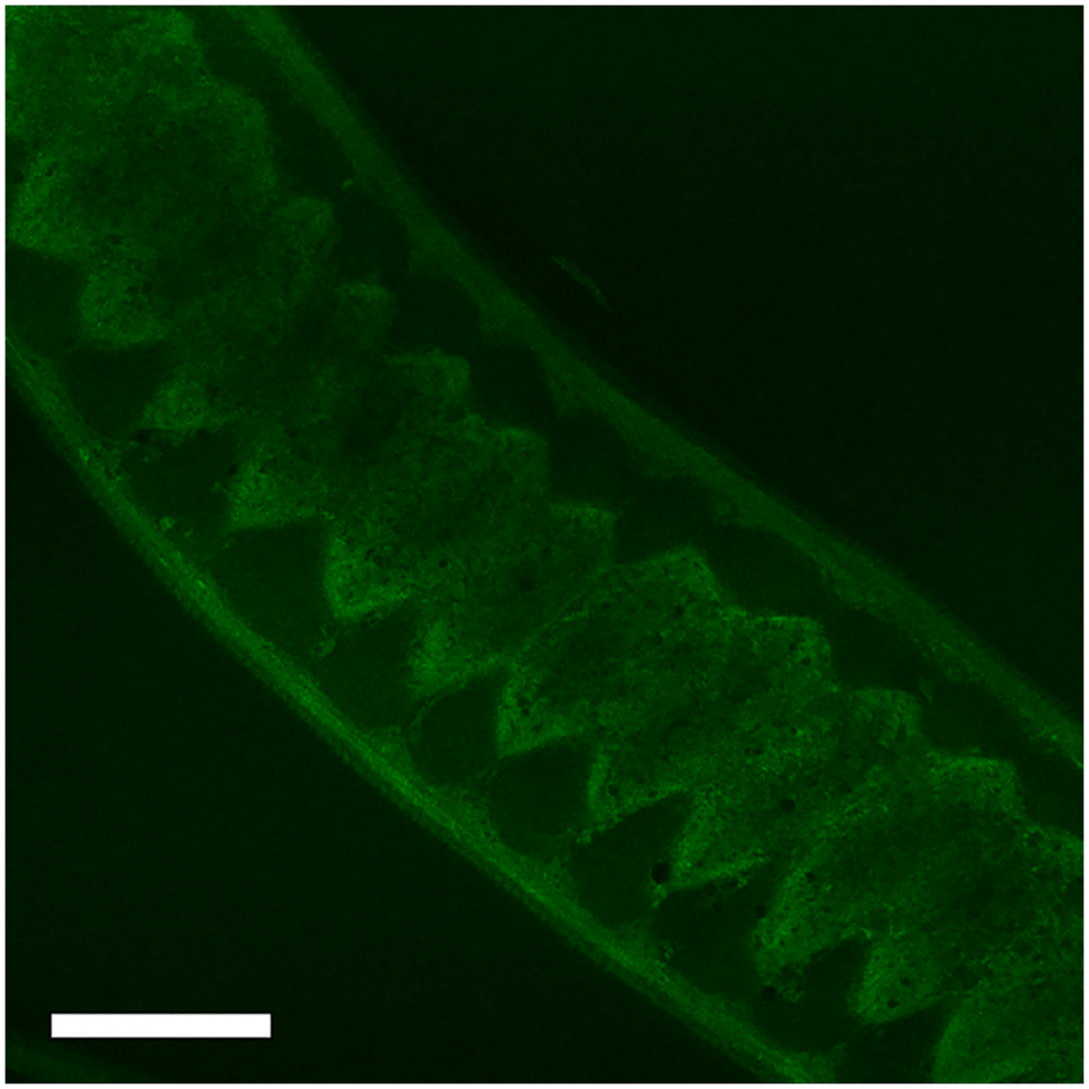
Confocal section through the thin anterior part of an adult worm of *Trichuris muris* with sealed oral cavity after incubation in agarose (1%) with 6-(*N*-(7-Nitrobenz-2-oxa-1,3-diazol-4-yl)amino)-6-Deoxyglucose (6-NBDG) (200 μM) for approximately 40 minutes. Despite the sealed oral cavity, 6-NBDG accumulated within the stichocytes and the connective membranes. Scale bar: 50 μm.

All *A*. *suum* with and without sealed oral cavities were alive at day 7. Glue-caps attached to a living worm at day 7, and separated from euthanized worms are shown in S3 Fig. No visible holes, disruption of glue-cap attachments, or tissue damage of *A*. *suum* were stereo microscopically observed during the 7-day period. As the experiment (including oral sealing of *T*. *muris*) had a time span of only 2 hours, Histoacryl was considered suitable for the purpose. During the experiment, the posterior end of *T*. *muris* was motile. The anterior part of *T*. *muris* with and without sealed oral cavities is shown in S4 Fig. The non-sealed worm had some 6-NBDG in the anterior part of the oesophagus. The glue has a crystal-like appearance that covers the most anterior part of the worm on both sides, hence the ingestion of 6-NBDG through the oral cavity is most likely precluded. Despite the oral sealing with glue and agarose, 6-NBDG accumulated within the stichocytes, with mean grey values increasing from 5.8 to 8.1 and 9.4 after 29 (Fig 9a) 44 (Fig 9b) and 56 min. (Fig 9c), respectively. As observed for *T*. *muris* with an open oral cavity (Fig 7), 6-NBDG also accumulated in the connecting membranes of worms with a sealed oral cavity (Fig 10), and in addition, a weak fluorescent signal was observed between 500–600 nm (i.e. the same emission wavelengths as 6-NBDG) in the posterior part of the intestine and the cloaca (S5 Fig).

## Discussion

We have demonstrated that adult *T*. *muris* are dependent on glucose for viability *in vitro*, and that the bacillary band does have an absorptive function in relation to the glucose analogue 6-NBDG. Furthermore, we have indicated that the accumulation of 6-NBDG within the worm may be independent of oral uptake. However, minor amounts of 6-NBDG passing through the oral cavity may not be completely excluded. Our results on adult worm glucose dependency are in contrast to those of Wimmersberger et al. 2013 [51], who reported that glucose (2%) did not improve the survival rates of L1 larvae of *T*. *muris*, harvested after the hatching of eggs induced by bacteria. However, the bacillary band develops as the worm matures, i.e. the number of pores increase throughout the development stages [31]. It is therefore possible that different life stages of *T*. *muris* are dependent on different types of nutrients, or that the L1 larvae are reliant on internally stored energy reserves (i.e. glycogen), as seen in other parasitic nematodes [52]. The absorption of 6-NBDG through the bacillary band suggests a unique adaptation of *Trichuris* spp. to the intracellular environment of the gut epithelium from where nutrients can be absorbed by the bacillary band, as speculated by Tilney et al. [31]. However, as we observed 6-NBDG in the anterior part of oesophagus (S4 Fig), oral ingestion may not be fully excluded. It is therefore possible that the adaptation to an intracellular environment is still on-going and that oral ingestion still plays a minor role in nutrition uptake for this parasitic nematode.

Only weak autofluorescence was observed in three adult *T*. *muris* prior to this study, however during the live-uptake studies (+/- 6-NBDG), autofluorescence of the bacillary band was detected (S2 and S3 Videos). The three worms tested prior to this study were not incubated in media devoid of glucose and it is therefore possible that the observed autofluorescence was due to a stress response related to glucose deprivation during the incubation time. However, the autofluorescence of the test worms (+6-NBDG) disappeared abruptly when 6-NBDG containing RPMI media was added. This could be related to the above, and that the excitation light in out-of-focus planes was absorbed by 6-NBDG when added, resulting in a decreased excitation in the focal plane. The abrupt disappearance of the autofluorescence was not detected in the control worms which may be in concordance with the above explanation.

We have further demonstrated an accumulation of 6-NBDG in the stichocytes and their connecting membranes. The connection between the bacillary band and the stichocytes has been debated for decades. Müller et al. [53] suggested that the bacillary cells secrete a substance which dissolves the host tissue, after which the digesta is absorbed and transported to the stichosomes through the connecting membrane, whereas Chitwood and Chitwood [54] described these membranes as solely supporting structures. However, Jenkins [34,55] found that the hypodermis of *T*. *suis* is relatively broad beneath the bacillary band and has a cellular structure which is cytochemically active. Hence, this author further speculated that the hypodermis was involved with the nematode’s own metabolism and may be used as storage centres for glycogen and lipids. In a later study, Hüttemann et al. [39] reported a cellular connection between the basal lamina surrounding the bacillary cells and the stichocytes. In our study, the origin of the connecting membranes (i.e. hypodermis and/or cellular connections between the bacillary cells and the stichocytes) was not further investigated, but as 6-NBDG accumulated within the stichocytes and the connecting membranes of worms with sealed oral cavities, it is most likely that 6-NBDG was absorbed by the bacillary band and reached the stichocytes through the connective membranes, as suggested by Müller et al. [53].

Several authors [39,56] have suggested that nutrients pass from the stichocytes into the digestive tract, as the presence of connecting ducts between these structures has been reported for *Trichinella spiralis* [57] and *Capillaria pterophylli* [58]. Sheffield [56] reported the presence of tube-like structures (i.e. canaliculi) within the stichocytes of *T*. *muris* and *T*. *vulpis*, and found similar vesicles in the stichosomes and oesophagus. Although unable to demonstrate a duct between the stichocytes and oesophagus, the author suggested that an intracellular system for the transport of material into the oesophageal lumen was present. A later study of *T*. *muris* confirmed the occurrence of vesicles with similar content in the stichocytes, pharynx and the pseudocel [39]. It was suggested that this observation was related to vesicular transport from the bacillary cells to the pharynx via the pseudocel and stichocytes. We were unable to demonstrate the collecting duct between the stichocytes and the digestive tract using confocal microscopy, but as a weak fluorescent signal was observed between –500–600 nm (i.e. the same emission wavelengths as 6-NBDG) in the posterior part of the intestine and the cloaca of worms with sealed oral cavities we speculate that such a connection is present. However, as the fluorescent intensity was very low, and absorption through the cloaca could not be excluded in this study, the connection between the stichocytes and the oesophagus still needs to be confirmed.

Based on our findings, we conclude that the bacillary band of *T*. *muris* has an absorptive function in relation to the glucose analogue 6-NBDG, which may be transported from the bacillary cells to the intestine via the connecting membranes and the stichocytes. Further studies on the function of the bacillary band may reveal a possible role in relation to absorption of anthelminthics.

## Supporting Information

S1 Fig Transmission image corresponding to S2 Video. Scale bar: 50μm.
(TIF)

S2 Fig Transmission image corresponding to S3 Video. Scale bar: 50μm.
(TIF)

S3 Fig Glue caps of Histoacryl. Glue caps of Histoacryl attached to (a) and separated from (b and c) adult *Ascaris suum* after 7-days incubation in RPMI media.
(TIF)

S4 Fig *Trichuris muris* with and without a sealed oral cavity. Transmission (a) and confocal image (b) of the anterior tip of an adult *Trichuris muris* without glue, and transmission image (c) of the anterior tip of an adult *Trichuris muris* with a glued oral cavity. Note the smooth surface of the worm (a and b) and some 6-(*N*-(7-Nitrobenz-2-oxa-1,3-diazol-4-yl)amino)-6-Deoxyglucose (6-NBDG) in the anterior part of the oesophagus (b) of the worm without glue, and the crystal-like appearance of the glue covering the whole anterior tip of the glued worm (c). Scale bar: 50μm.
(TIF)

S5 Fig The posterior part of an adult *Trichuris muris* with a sealed oral cavity. Transmission (a) and confocal image (b and c) of the posterior part of the intestine and the cloaca of an adult *Trichuris muris* with sealed oral cavity. Note the weak fluorescent signal in the intestinal tract. Scale bar: 50 μm.
(TIF)

S1 Video Z-stack through the bacillary band of an adult *Trichuris muris*. Fluorescence from 6-NBDG and Alexa Fluor 633 is shown in green and red respectively. The vertical distance between the images is 0.2 μm. Scale bar: 10 μm.
(ZIP)

S2 Video Live-uptake of 6-NBDG through the bacillary band of *Trichuris muris*. The glucose analogue 6-(*N*-(7-Nitrobenz-2-oxa-1,3-diazol-4-yl)amino)-6-Deoxyglucose (6-NBDG) was added at 00:00:35:00, with time displayed (upper right) as hour:min:sec:msec. Before addition, some autofluorescence was seen from the bacillary band. Immediately after addition, this fluorescence decreased to almost half of the intensity. After the initial decrease, the intensity increased in mean grey values from a minimum of 1.7 to a maximum of 5.8 after 20 minutes. The length of the video is 20 minutes.
(ZIP)

S3 Video Control of experiment shown in S2 Video. RPMI media without 6-(*N*-(7-Nitrobenz-2-oxa-1,3-diazol-4-yl)amino)-6-Deoxyglucose (6-NBDG), and with DMSO (0.2 v/v%) is added. Immediately after addition, the fluorescence did not decrease abruptly to almost half of the intensity as in the test worms. The intensity from the bacillary band did not increase but showed a small decrease in mean grey values from 4.2 to 3.4 during a time period of 20 minutes.
(ZIP)
