# Supplementary figures and images for: Glucose Absorption by the Bacillary Band of Trichuris muris
Source: PLoS Negl Trop Dis. 2016 Sep 2;10(9):e0004971. doi: 10.1371/journal.pntd.0004971 (PMC5010283; doi:10.1371/journal.pntd.0004971)

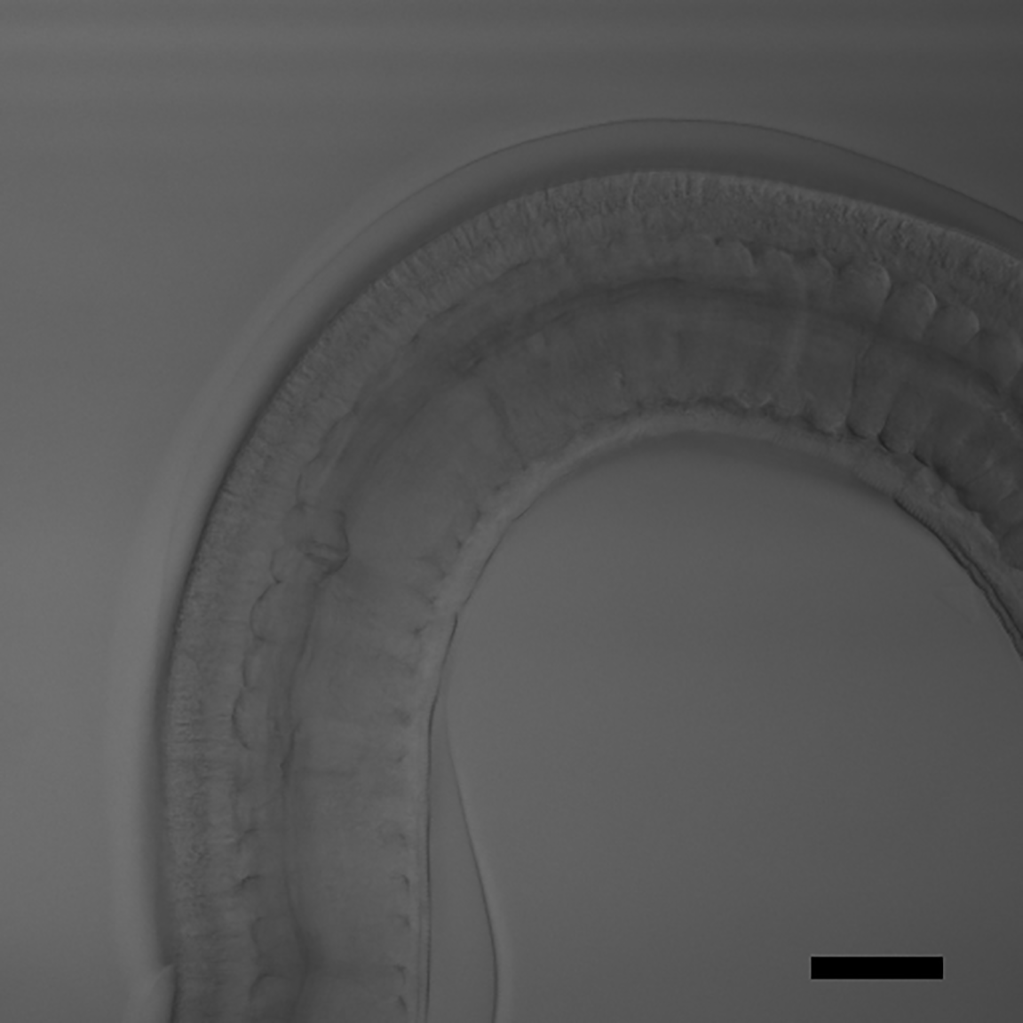

Supplement: S1 Fig — Scale bar: 50μm. (TIF) [file pntd.0004971.s001.tif]

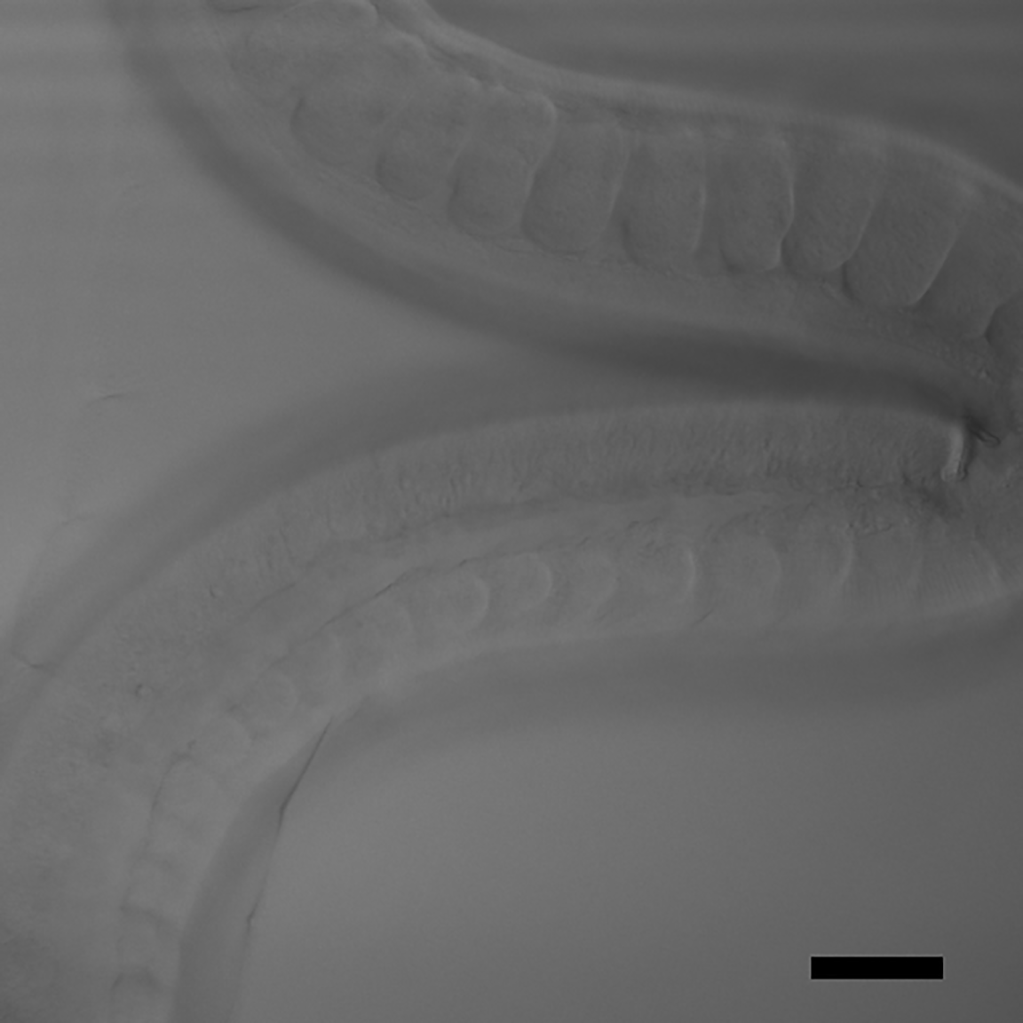

Supplement: S2 Fig — Scale bar: 50μm. (TIF) [file pntd.0004971.s002.tif]

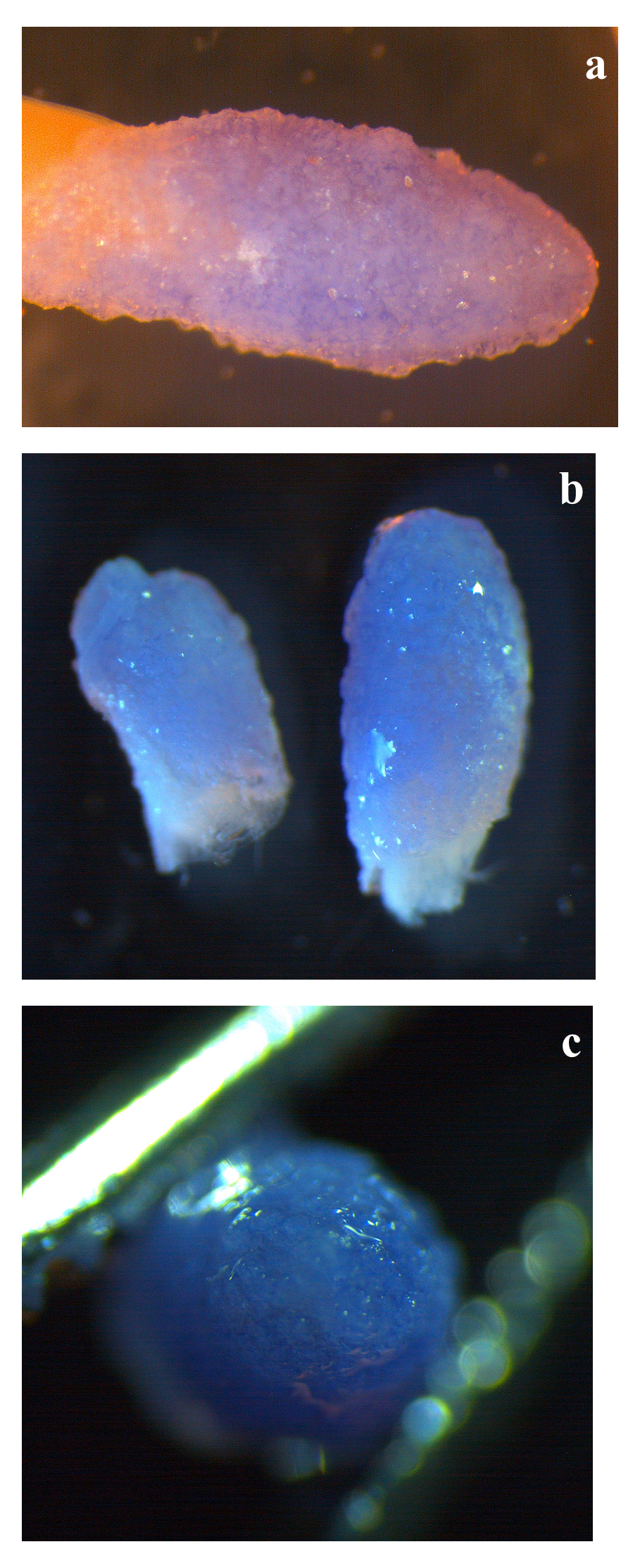

Supplement: S3 Fig — Glue caps of Histoacryl attached to (a) and separated from (b and c) adult Ascaris suum after 7-days incubation in RPMI media. (TIF) [file pntd.0004971.s003.tif]

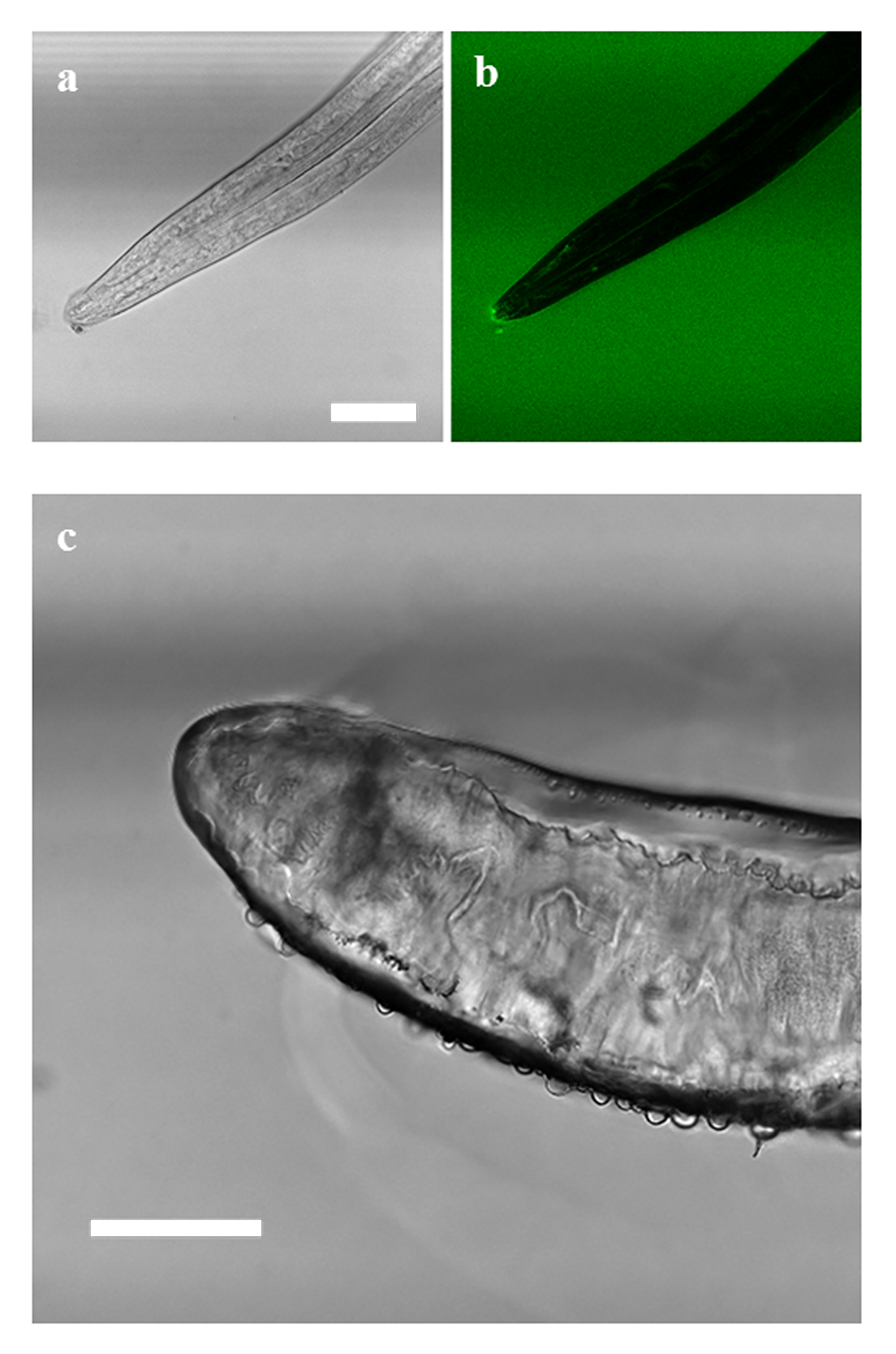

Supplement: S4 Fig — Transmission (a) and confocal image (b) of the anterior tip of an adult Trichuris muris without glue, and transmission image (c) of the anterior tip of an adult Trichuris muris with a glued oral cavity. Note the smooth surface of the worm (a and b) and some 6-(N-(7-Nitrobenz-2-oxa-1,3-diazol-4-yl)amino)-6-Deoxyglucose (6-NBDG) in the anterior part of the oesophagus (b) of the worm without glue, and the crystal-like appearance of the glue covering the whole anterior tip of the glued worm (c). Scale bar: 50μm. (TIF) [file pntd.0004971.s004.tif]

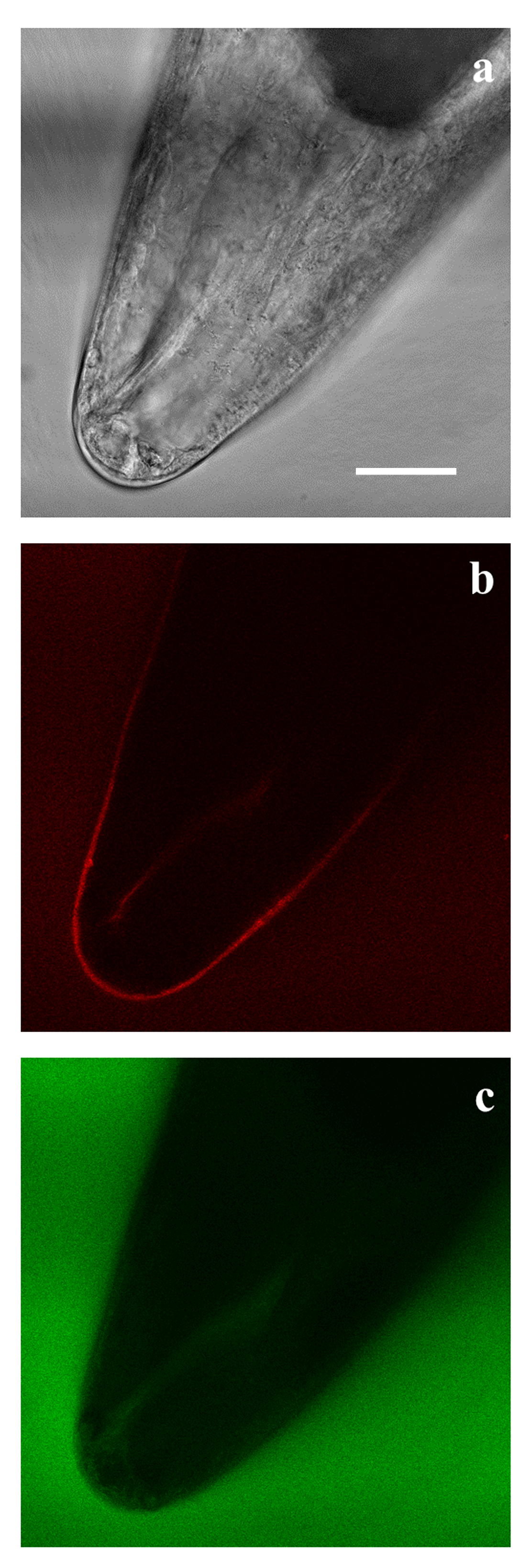

Supplement: S5 Fig — Transmission (a) and confocal image (b and c) of the posterior part of the intestine and the cloaca of an adult Trichuris muris with sealed oral cavity. Note the weak fluorescent signal in the intestinal tract. Scale bar: 50 μm. (TIF) [file pntd.0004971.s005.tif]
